# Clinical Significance of Indeterminate QuantiFERON-TB Gold Plus Assay Results in Hospitalized COVID-19 Patients with Severe Hyperinflammatory Syndrome

**DOI:** 10.3390/jcm10050918

**Published:** 2021-02-26

**Authors:** Xavier Solanich, Miguel Fernández-Huerta, Celeste Basaez, Arnau Antolí, Gemma Rocamora-Blanch, Xavier Corbella, Miguel Santin, Fernando Alcaide

**Affiliations:** 1 Department of Internal Medicine, Bellvitge University Hospital, 08907 L’Hospitalet de Llobregat, Barcelona, Spain; aantolig@bellvitgehospital.cat (A.A.); grocamora@bellvitgehospital.cat (G.R.-B.); xcorbella@bellvitgehospital.cat (X.C.); 2 Bellvitge Biomedical Research Institute (IDIBELL), 08907 L’Hospitalet de Llobregat, Barcelona, Spain; mfernandezh@bellvitgehospital.cat (M.F.-H.); msantin@bellvitgehospital.cat (M.S.); falcaide@bellvitgehospital.cat (F.A.); 3 Department of Microbiology, Bellvitge University Hospital, 08907 L’Hospitalet de Llobregat, Barcelona, Spain; 4 Biochemistry Department, Hospital Interzonal General de Agudos Evita de Lanús, 1826 Lanús, Argentina; celebasaez@gmail.com; 5 School of Medicine, Universitat Internacional de Catalunya, 08017 Barcelona, Spain; 6 Department of Infectious Diseases, Bellvitge University Hospital, 08907 L’Hospitalet de Llobregat, Barcelona, Spain; 7 Department of Infectious Diseases, University of Barcelona, 08907 L’Hospitalet de Llobregat, Barcelona, Spain

**Keywords:** QuantiFERON-TB Gold Plus, indeterminate, COVID-19, SARS-CoV-2, corticosteroids

## Abstract

Performance of the QuantiFERON-TB Gold Plus (QFT-Plus) assay could be affected by conditions of immune dysregulation. Little is known about the reliability of QTF-Plus in COVID-19 patients. Our aim was to determine the prevalence and the factors related to an indeterminate QFT-Plus test in COVID-19 hospitalized patients, and to analyze its relationship with in-hospital mortality. A retrospective analysis of all hospitalized COVID-19 patients on whom a QTF-Plus assay was performed in a tertiary care public hospital during the first epidemic wave in Spain (March–April 2020). Out of a total of 96 patients included, 34 (35.4%) had an indeterminate result, in all cases due to a lack of response in the mitogen control. Factors related to COVID-19 severity, such as higher lactate dehydrogenase (LDH) (odds ratio [OR] 1.005 [95% confidence interval [CI] 1.002–1.008]) and previous administration of corticosteroids (OR 4.477 [95% CI 1.397–14.345]), were independent predictors for indeterminate QFT-Plus assay. Furthermore, indeterminate results were more frequent among COVID-19 patients who died during hospitalization (29.1% vs. 64.7%; *p* = 0.005). We conclude that QFT-Plus assay yielded an unexpected, high prevalence of indeterminate results in severe COVID-19 patients. Factors related to worse COVID-19 outcome, such as LDH, as well as corticosteroid use before the QFT-Plus assay, seem to be predictors for an indeterminate result. The role of an indeterminate QFT-Plus result in predicting COVID-19 severity and mortality should be evaluated.

## 1. Introduction

In December 2019, an emerging disease (COVID-19), caused by a newly identified human coronavirus, was first recognized in Wuhan, China, and spread worldwide [1,2]. The WHO declared the COVID-19 epidemic to be a pandemic on 12 March 2020 [3], and it continues to spread globally today, causing considerable morbimortality and economic damage.

Multiple drugs have been repurposed to treat severe SARS-CoV-2 infections presenting with systemic hyperinflammatory syndrome, some of them having an immunosuppressive effect such as corticosteroids, tocilizumab, anakinra, etc. [4,5,6,7]. Therefore, reactivation of several dormant infections, including latent tuberculosis infection (LTBI), is of concern among COVID-19-treating physicians. In this regard, interferon-gamma release assays (IGRAs), such as the QuantiFERON-TB Gold Plus (QFT-Plus; Qiagen, Germany) [8] assay, have been unsystematically performed for LTBI screening in some severe COVID-19 patients, before or during immunosuppressive therapy.

QFT-Plus is based on the detection of interferon-gamma (IFN-γ) released by a T-cell-mediated immune response following in vitro stimulation of human whole-blood by antigens specific to the Mycobacterium tuberculosis complex [8]. Additionally, the QFT-Plus assay includes a mitogen-based control designed to nonspecifically elicit a T-cell response and thus infer the immunological fitness of the studied individuals. It is well known that active inflammation or immunosuppressive drug use is associated with QFT-Plus indeterminate results [8,9].

During the first months of the COVID-19 pandemic, microbiologists observed a surprisingly high rate of QFT-Plus indeterminate results in patients hospitalized with COVID-19 at our hospital. At that time, there was no information about this finding and, nowadays, there is still little information about how COVID-19 itself and the immunosuppressive drugs can impact QFT-Plus performance [10]. In addition, it is not known if an indeterminate QFT-Plus result might have a relation to COVID-19 severity and mortality.

Our aim was to determine the prevalence and the factors related to an indeterminate QFT-Plus test in COVID-19 hospitalized patients, and to analyze its relationship with in-hospital mortality.

## 2. Materials and Methods

### 2.1. Study Design and Participants

The study was conducted at the Bellvitge University Hospital (BUH), a 750-bed tertiary-care public hospital for adults in Barcelona, Spain. BUH is the reference hospital for 2 million inhabitants with high-complexity diseases from the Southern area of Catalonia.

We performed a retrospective study of all hospitalized patients with COVID-19 from March to April 2020. SARS-CoV-2 infection was confirmed by RT-PCR in all patients on whom the QFT-Plus test was performed. Demographic, epidemiological, laboratory and clinical data were also collected. Laboratory tests were determined within 24 hours before or after the QFT-Plus test. The QFT-Plus indeterminate results were defined according to the manufacturer´s criteria. Nil value comes from the negative control tube that contains no additives, and it is used to determine if the patient has a pre-existing immune disturbance which could cause a false-positive or false-negative reading on the test (that is, nil > 8 UI/mL or nil-corrected mitogen value < 0.5 IU/mL) [8]. Mild hypoxemia was defined as PF (PaO_2_/FiO_2_) ratio < 300 and/or SF (SpO_2_/FiO_2_) ratio < 357; moderate hypoxemia was defined as PF ratio < 200 and/or SF ratio < 214; and severe hypoxemia was defined as PF ratio < 100 and/or SF ratio < 89) [11]. In accordance with the WHO 8-point ordinal scale [12], a moderate disease was defined as hospitalized patients with or without supplementary oxygen; and a severe disease was considered in patients with non-invasive ventilation or high-flow oxygen, intubation and mechanical ventilation, additional organ support, renal replacement treatment or extracorporeal membrane oxygenation (ECMO), or death. We calculated the WHO 8-point ordinal scale corresponding to the same day that the QFT-Plus assay was performed for each participant. Treatments specifically used to treat COVID-19 at any time during admission, and immunosuppressive treatments administered before the QFT-Plus test was performed were also analyzed. According to the March to April 2020 BUH guidelines, corticosteroids were administered as methylprednisolone iv 125 mg daily on 3 consecutive days, and iv tocilizumab as a single dose (600 mg if ≥75 kg or 400 mg if <75 kg). Length of stay from QFT-Plus assay until hospital discharge or death was also recorded.

### 2.2. Statistical Analysis

Results from continuous variables are presented as median and interquartile range (IQR), and categorical data as absolute frequencies and percentages. Comparisons of the cohorts were made using a chi-square test or Fisher’s exact test for categorical variables and a Mann–Witney U test for continuous variables. Multivariable logistic regression analysis using the stepwise method was performed to assess factors related to an indeterminate QFT-Plus assay result for not inter-correlated and univariate *p*-value ≤ 0.1 co-variables, since we do not know which ones may be the most relevant due to the lack of previous studies in patients with COVID-19. Survival analysis was conducted using Cox univariate regression and Kaplan–Meier curves to assess the impact of an indeterminate QFT-Plus assay on the COVID-19 in-hospital mortality. Statistical significance was defined as *p*-value < 0.05 and we also used odds ratios (OR) or hazard ratios (HR) and their 95% confidence intervals (CI). Calculations were performed with the statistical package SPSS version 19 (IBM Corp., USA).

## 3. Results

The QFT-Plus test was performed on 96 (6.8%) of a total of 1403 patients admitted due to COVID-19 during the study period. Main comorbidities, clinical status, laboratory tests and treatment characteristics are summarized in Table 1. In all cases, the QFT-Plus assay was indicated for the screening of LTBI in patients receiving or potentially going to receive immunosuppressive therapy to treat severe COVID-19. Finally, of the overall 96 cases, 66 (68.8%) received corticosteroids and/or tocilizumab, and the rest did not. The QFT-Plus test was performed with a median of 12 (IQR 10–16) days from the onset of COVID-19 symptoms. Of the overall 96 patients tested, 54 (56.3%) were negative, 8 (8.3%) were positive, and 34 (35.4%) were indeterminate. Any patient developed tuberculosis after a median follow-up period of 74 days (IQR 61–78). All indeterminate results were due to a lack of response to the mitogen control.

There was a significant association between an indeterminate QFT-Plus result and lymphopenia (0.64 [0.48–1.15] vs. 0.88 [0.64–1.27] × 109; *p* = 0.017), leukocytosis (8.6 [6.3–13.9] vs. 6.8 [5.6–9.6] × 109; *p* = 0.016), and neutrophilia (7.3 [5.4–12.4] vs. 5.5 [3.5–8.3] × 109; *p* = 0.002). Indeterminate QFT-Plus assay results were associated with higher levels of lactate dehydrogenase (LDH) (412 [330–517] vs. 317 [265–424] U/L; *p* = 0.003) and D-dimer (636 [353–2913] vs. 361 [250–946] µg/L; *p* = 0.025), as well as hypoxemia (*p* = 0.015). Additionally, corticosteroid use prior to the QFT-Plus test was significantly higher in the indeterminate subset (*n* = 10/34 [29.4%] vs. *n* = 6/62 [9.7%]; *p* = 0.013). Significantly, five of the 34 (14.7%) patients who did not receive immunosuppressive treatment prior to testing showed indeterminate results. However, no association between indeterminate QFT-Plus and age, gender, comorbidities or previous days of COVID-19 was found. In the adjusted analysis, higher LDH (OR 1.005 [95% CI 1.002–1.008]) and corticosteroid use before performing the QFT-Plus test (OR 4.477 [95% CI 1.397–14.345]) remained independently associated with an indeterminate test (Table 1).

**Figure 1:**
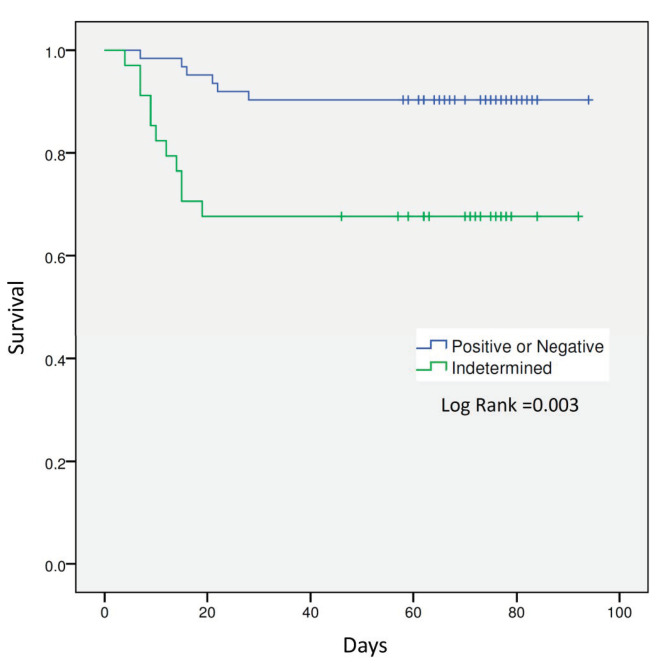
Kaplan–Meier survival curves for patients with interpretable (positive or negative) and indeterminate QFT-Plus assay results.

Seventeen (17.7%) patients died during hospitalization. In this respect, a significant association was found between mortality and an indeterminate QFT-Plus assay result (*n* = 23/79 [29.1%] vs. *n* = 11/17 [64.7%]; *p* = 0.005). Patients with an indeterminate QFT-Plus test showed four times more risk for in-hospital mortality (HR 4.025 [95% CI 1.486–10.903]) than those patients with interpretable results (Figure 1).

## 4. Discussion

The present study shows an unexpected, high prevalence of indeterminate results in hospitalized patients with COVID-19. Factors related to COVID-19 clinical severity such as higher levels of LDH and corticosteroid use before the performance of the QFT-Plus assay seem to be related to an indeterminate result. Furthermore, a significant association between an indeterminate QFT-Plus result and higher in-hospital mortality has also been found.

The sensitivity of IGRAs in the detection of LTBI can be affected by conditions of immune dysregulation. Our research group previously assessed IGRAs in several immune-related disorders, detecting a percentage of indeterminate results of 2.1% in pre-transplant cirrhotic patients [13], 7.7% in patients with corticosteroids who are going to receive anti-TNF alpha treatments [14], or 9.5% in HIV-positive patients without antiretroviral treatment and a CD4 count <100 cells/mm^3^ [15]. Of great concern, the present study found that 35.4% of the hospitalized COVID-19 patients studied had an indeterminate QFT-Plus assay result. This proportion is consistent with the 36.4% rate reported by Torre et al. in a recent work from an Italian series [10]. So, the frequency of indeterminate results might be significantly high in COVID-19 patients.

In our study, the QFT-Plus test was mostly performed in severe COVID-19 patients hospitalized after 10 days of symptoms onset, and the results obtained are representative of the COVID-19 inflammatory phase [16]. According to previous reports, COVID-19 patients at this inflammatory stage of the disease show high plasma proinflammatory cytokines levels (IL-1b, IL-2, IL-4, IL-7, IL-10, MCP-1, GCSF, MIP-1A, TNF-α, IFN-γ and IP-10) [17,18]; however, this fact should not alter the accuracy of the QFT-Plus test since indeterminate results were not due to a high IFN-γ concentration in the nil tube (>8 UI/mL) [8].

Therefore, we investigated those potential factors which may cause indeterminate QFT-Plus results due to IFN-γ concentrations in the mitogen-based control below the limit established by the test manufacturer (that is, nil-corrected mitogen value < 0.5 IU/mL). Accordingly, our rationale was that COVID-19 patients at severe stages might suffer for some kind of immunosuppressant condition. According to our data, two groups of factors might be associated with indeterminate results: firstly, some determinants that have been previously related to worse COVID-19 outcome (lymphopenia, leukocytosis, neutrophilia, LDH, D-dimer, hypoxemia) [17,19] and, secondly, the use of immunosuppressive treatments administered before the QFT-Plus assay, especially corticosteroids.

Corticosteroids and other immunosuppressive drugs are well known to be the cause of indeterminate QFT-Plus assay results [14]. However, it should be noted that the indeterminate results rate remained high (14.7%) even in the subset of our studied patients who had not received any immunosuppressive treatment prior to test, highlighting COVID-19 itself as a determining factor of an indeterminate result.

SARS-CoV-2 has evolved several mechanisms to impair host immune response, including the inhibition of IFN induction and signaling. In fact, impaired IFN-I/III signatures have been related to a persistent blood viral load and an inflammatory disturbance that leads to a worse COVID-19 prognosis [18,20]. Nevertheless, there are conflicting data regarding the role of type II IFNs such as IFN-γ. In several studies, elevation of multiple inflammatory cytokines, including IFN-γ, has been related to a more severe COVID-19 [18,21]. On the other hand, recent data show that COVID-19 has the ability to induce an early and profound suppression of T-cell IFN-γ production [22]. Possibly, these data suggest that timing is the key, as IFN-γ could be protective early in a disease but later could become pathologic and increase as a consequence of multiple stimuli received by the hyperinflammatory cytokine storm. In clinical practice, there is a lack of diagnostic tools to evaluate the failure of host protective immunity while suffering from COVID-19. In this respect, the QFT-Plus test might be a standardized and accessible functional immunoassay to study the IFN- γ host immunity.

Worryingly, the rate of patients with an indeterminate QFT-Plus assay result was higher in patients who died during admission (64.7% vs. 29.1%). Several factors have been related to in-hospital mortality in COVID-19 [14,16], but there is scarce information regarding the role of an indeterminate QFT-Plus result as a marker of severity or death.

This preliminary investigation of COVID-19 patients with an indeterminate QFT-Plus assay result has several limitations that deserve further comment. We performed a retrospective analysis so confounders could be overlooked and missing data might have altered some results. Only 6.8% of COVID-19 patients admitted to hospital were tested for QFT-Plus assay, and therefore this is a highly selected group with potential for bias. Furthermore, the QFT-Plus assay was unsystematically performed in hospitalized patients with COVID-19, although in most cases was done in those who transited into inflammatory stages receiving or going to receive immunosuppressive agents such as corticosteroids and/or tocilizumab. Therefore, the present study does not allow us to assess the QFT-Plus assay usefulness in patients at earlier stages of the disease. The number of COVID-19 patients on whom a QFT-Plus assay was performed is relatively low to draw solid conclusions about its role in predicting clinical outcomes of the disease.

## 5. Conclusions

The indeterminate QFT-Plus assay results show an unexpected, high prevalence in hospitalized COVID-19 patients with host-immune hyperinflammatory syndrome, losing reliability in this context. Factors related to worse COVID-19 outcome such as LDH, as well as corticosteroid use before the QFT-Plus assay, seem to be predictors for an indeterminate result. Furthermore, indeterminate QFT-Plus results were found in a higher proportion in those COVID-19 patients who died. Therefore, further and larger studies are needed to assess the real significance of indeterminate QFT-Plus test results on COVID-19 clinical outcomes.

## Figures and Tables

**Table 1:** Main characteristics of 96 COVID-19 hospitalized patients according the QuantiFERON-TB Gold Plus (QFT-Plus) test result.

	Indeterminate QTF-Plus (*n* = 34)	Interpretable QTF-Plus (*n* = 62)		Unadjusted Model		Adjusted Model	
			*p*-Value	OR (95%CI)	*p*-Value	OR (95%CI)	*p*-Value
**Gender (male)**	22 (64.7%)	45 (72.6%)	0.422	0.693 (0.282–1.700)	0.422		
**Age (years)**	70.5 (56.9–76.2)	63.7 (49.2–74.9)	0.237	1.020 (0.990–1.051)	0.203		
**Comorbidities**							
Hypertension	16 (47.1%)	34 (54.8%)	0.466	0.732 (0.316–1.694)	0.466		
Dyslipidemia	19 (55.9%)	28 (45.2%)	0.315	1.538 (0.663–3.569)	0.315		
Diabetes	11 (32.4%)	12 (19.4%)	0.154	1.993 (0.766–5.182)	0.154		
Cardiovascular disease	7 (20.6%)	12 (19.4%)	0.885	1.080 (0.381–3.066)	0.885		
Lung disease	6 (17.6%)	9 (14.5%)	0.686	1.262 (0.408–3.906)	0.686		
Immunocompromised patient	4 (11.8%)	3 (4.8%)	0.212	2.622 (0.551–12.480)	0.212		
**Candidate for invasive measures (yes)**	25 (73.5%)	52 (83.9%)	0.224	0.534 (0.193–1.480)	0.224		
**COVID-19 onset to admission (days)**	7.5 (5.75–12.25)	8 (7–10.25)	0.401	1.013 (0.928–1.105)	0.778		
Laboratory tests (*)							
Leukocytes (×10^9^/L)	8.6 (6.3–13.9)	6.8 (5.6–9.6)	0.016	1.131 (1.024–1.248)	0.015		
Neutrophils (×10^9^/L)	7.3 (5.4–12.4)	5.5 (3.5–8.3)	0.002	1.147 (1.032–1.274)	0.011		
Lymphocytes (×10^9^/L)	0.64 (0.48–1.15)	0.88 (0.64–1.27)	0.017	0.287 (0.105–0.783)	0.015		
Serum ferritin (μg/L) (*n* = 92)	1756 (1081–2574)	1444 (692–2233)	0.159	1.000 (1.000–1.001)	0.201		
LDH (U/L) (*n* = 95)	412 (330–517)	317 (265–424)	0.003	1.005 (1.001–1.008)	0.005	1.005 (1.002–1.008)	0.003
C-reactive protein (mg/L)	79 (29–197)	87 (27–160)	0.654	1.002 (0.997–1.006)	0.447		
IL-6 (ng/L),(*n* = 52)	100 (20–1044)	99 (55–193)	0.955	1.001 (1.000–1.012)	0.130		
Troponin (ng/L), (*n* = 69)	10.0 (7.0–25.0)	10.5 (6.0–18.2)	0.546	1.000 (0.991–1.008)	0.926		
D-dimer (μg/L), (*n* = 93)	636 (353–2913)	361 (250–946)	0.025	1.000 (1.000–1.001)	0.016		
**Hypoxemia**			0.015				
None	4 (11.8%)	21 (33.8%)		-			
Mild	6 (17.6%)	20 (32.3%)		1.557 (0.386–6.423)	0.536		
Moderate	22 (64.7%)	20 (32.3%)		5.775 (1.690–19.734)	0.005		
Severe	2 (5.9%)	1 (1.6%)		10.5 (0.758–145.359)	0.079		
**COVID-19 onset to QFT-Plus (days)**	11 (9–18)	12 (10-16)	0.797	1.005 (0.937–1.077)	0.893		
**Severe WHO 8-OS on QFT-time**	9 (26.5)	5 (8.1)	0.015	4.104 (1.248–13.491)	0.015		
**IS prior to QFT-Plus**							
None	5 (14.7%)	28 (45.1%)	0.003	0.209 (0.072–0.612)	0.003		
Corticosteroids	10 (29.4%)	6 (9.7%)	0.013	3.889 (1.270–11.912)	0.013	4.477 (1.397–14.345)	0.012
IL-6 blockage	3 (8.8%)	5 (8.1%)	0.898	1.103 (0.247–4.928)	0.898		
Corticosteroids and IL-6 blockage	16 (47.1%)	23 (37.1%)	0.342	1.507 (0.646–3.519)	0.342		

Qualitative data are expressed as a number (percentage), and quantitative data are expressed as a median (interquartile range). Abbreviations: OR (95%CI), odds ratio (95% confidence interval); IL-6, interleukin-6; QFT, QuantiFERON-TB Gold Plus; LDH, lactate dehydrogenase; IS, immunosuppressive drugs; WHO 8-OS, World Health Organization 8-point Ordinal Scale. (*) Laboratory tests when the QFT-Plus assay was performed.

## Data Availability

The data sets generated and analyzed during the current study are available on request to the corresponding author upon reasonable request.
